# The Type III Secreted Effector DspE Is Required Early in *Solanum tuberosum* Leaf Infection by *Pectobacterium carotovorum* to Cause Cell Death, and Requires Wx_(3–6)_D/E Motifs

**DOI:** 10.1371/journal.pone.0065534

**Published:** 2013-06-03

**Authors:** Clifford S. Hogan, Beth M. Mole, Sarah R. Grant, David K. Willis, Amy O. Charkowski

**Affiliations:** 1 Department of Plant Pathology, University of Wisconsin-Madison, Madison, Wisconsin, United States of America; 2 Department of Biology and Curriculum in Molecular Biology and Genetics, University of North Carolina at Chapel Hill, Chapel Hill, North Carolina, United States of America; 3 Vegetable Crops Research Unit, United States Department of Agriculture – Agricultural Research Service, Madison, Wisconsin, United States of America; Charité-University Medicine Berlin, Germany

## Abstract

*Pectobacterium* species are enterobacterial plant-pathogens that cause soft rot disease in diverse plant species. Unlike hemi-biotrophic plant pathogenic bacteria, the type III secretion system (T3SS) of *Pectobacterium carotovorum* subsp. *carotovorum* (*P. carotovorum*) appears to secrete only one effector protein, DspE. Previously, we found that the T3SS regulator HrpL and the effector DspE are required for *P. carotovorum* pathogenesis on leaves. Here, we identified genes up-regulated by HrpL, visualized expression of *dspE* in leaves, and established that DspE causes host cell death. DspE required its full length and WxxxE-like motifs, which are characteristic of the AvrE-family effectors, for host cell death. We also examined expression in plant leaves and showed that *hrpL* is required for the expression of *dspE* and *hrpN*, and that the loss of a functional T3SS had unexpected effects on expression of other genes during leaf infection. These data support a model where *P. carotovorum* uses the T3SS early in leaf infection to initiate pathogenesis through elicitation of DspE-mediated host cell death.

## Introduction

*Pectobacterium carotovorum* is a necrotrophic Enterobactericeae pathogen that causes soft rot disease on plant species from over 24 orders of angiosperms, including important crops in the Solanaceae and Brassicaceae. It is a ubiquitous pathogen that survives in soil, surface and ground water, and it has been associated with a variety of invertebrates [1], [2], [3], [4]. *P. carotovorum* attacks plant tissue by secreting an array of plant cell wall degrading enzymes through the type II secretion system [5], [6], [7], [8], [9], [10]. It also has a type III secretion system (T3SS) that is required for pathogenesis in leaves [11].

The genes encoding the T3SS typically lie in clusters of structural and regulatory genes called *hrp* (*h*ypersensitive *r*esponse and *p*athogenicity) that tend to be flanked by genes encoding secreted effectors and their chaperones [12], [13]. In plant pathogenic Enterobacteriaceae, such as *P. carotovorum*, and in the plant pathogen *Pseudomonas syringae,* the alternate sigma factor HrpL is the main regulator of genes in the *hrp* cluster and in these bacteria, HrpL regulates expression of all known T3-secreted effectors [14], [15], [16], [17], [18], [19].

Numerous categories of T3 effectors have been described in phytopathogenic bacteria. Unlike other T3 effectors, AvrE is widespread and found in *Pseudomonas*, *Pantoea*, *Dickeya*, *Pectobacterium*, and *Erwinia*. AvrE from *Pseudomonas syringae* blocks pathogen induced callose deposition on the plant cell wall to enhance virulence on host plants and AvrE causes cell death in leaves [20]. The AvrE-family effectors from *Erwinia amylovora* (DspE) and *Pantoea stewartii* (WtsE) suppress salicylic acid (SA)-mediated host defenses and also cause cell death in leaves [20], [21], [22], [23], and these effectors are essential for both *E. amylovora* and *Pantoea stewartii* pathogenesis [23], [24]. There is no evidence that *P. carotovorum* can suppress SA-mediate host defenses or callose deposition [11]. Mutagenesis experiments suggest that DspE is the only effector encoded by *P. carotovorum* and that DspE is required by the necrotrophic *P. carotovorum* to induce cell death on plant leaves [11].

In this work, we searched for additional HrpL regulated genes with a promoter-trap screen, but found none outside of the T3SS cluster, further supporting the hypothesis that *P. carotovorum* encodes a single effector, DspE. We found that the *hrpL* gene is required *in planta* for expression of *dspE* and *hrpN* and that a functional T3SS is required for expression of several additional genes, including the virulence factor gene *pelB*. To further define the role of the effector protein DspE in *P. carotovorum* pathogenesis, we used site-directed mutagenesis to identify residues critical for DspE function. We found that DspE alone can cause cell death and demonstrated that WxxxD/E motifs are important for this function, which supports the hypothesis that *P*. *carotovorum* pathogenesis in leaf tissue is initiated by DspE-mediated host cell death.

## Materials and Methods

### Bacterial Strains and their Growth Conditions

Bacterial strains and plasmids used in this study are listed in Table S1. *Escherichia coli* and *Pectobacterium carotovorum* strains were grown from single colonies in Luria-Bertani (LB) broth or 2xYT broth in a 250 rpm shaking incubator at 37°C or 28°C or maintained on LB plates solidified with 1.5% BD Bacto-agar, containing appropriate antibiotics. *Agrobacterium tumefaciens* strains were grown from single colonies in LB broth in a shaking incubator at 28°C or maintained on LB plates. The compositions of LB medium, M9 minimal medium, 2xYT media are published in Sambrook and Russell [25]. The minimal medium for *hrpL* repression was described in Chang et al. [26].

### Confocal Microscopy

Cultures were grown over night in LB, washed twice in 10 mM MgCl_2_ and re-suspended at an OD_600_ of 1.0 and infiltrated into leaves of 4–5 week old *A. thaliana* Col-0 using a needleless syringe. Potato leaves were not used because they were too thick to properly mount on microscope slides and visualize with confocal microscope. A Zeiss confocal microscope was used to visualize fluorescent bacteria *in planta* and images were captured and viewed with LSM image browser software (Zeiss). The experiment was repeated three times and representative samples are shown.

### DNA Manipulation, Transformation, and Sequencing

A FastDNA® SPIN for Soil Kit (MP Biomedicals, LLC, Solon, Ohio, U.S.A.) with a homogenization time of 5 seconds in a Mini Beadbeater™ (Biospec Products, Inc., Bartlesville, OK, U.S.A.) was used to isolate DNA from *P. carotovorum* grown overnight in LB. Appropriate Qiagen (Valencia, CA, U.S.A.) kits were used for isolation of DNA fragments from agarose gels or polymerase chain reaction (PCR) solutions. Platinum *Taq* DNA polymerase High Fidelity and Gateway vectors (Invitrogen, Carlsbad, CA, U.S.A.) were used to amplify and clone DNA, following the manufacturer’s protocols. All clones obtained were confirmed by DNA sequencing with an ABI 3730xl capillary electrophoresis sequencer (Applied Biosystems, Foster City, CA, U.S.A.) at the University of Wisconsin Biotechnology Center DNA Sequencing Facility or the University of North Carolina-Charlotte Genome Analysis Facility. Oligonucleotides were purchased from The University of Wisconsin Biotechnology Center DNA Synthesis Laboratory. Transformation of *A. tumefaciens* and *E. coli* DH5α-E™ was conducted by electroporation in 1 mm cuvettes using a GenePulser Xcell™ (Bio-Rad Laboratories, Hercules, CA, U.S.A.) apparatus set to 200 Ω and 1.8 kV. Transformation of *E. coli* DH5α™-T1^R^ was conducted by a heat shock protocol following the manufacturer’s protocols.

### Plasmid Construction

pBAD::*hrpL*WPP14 construction: *hrpL* was PCR amplified using Pfu (Stratagene) then cloned into pCFS40 as described in Chang et al. [26]. pBAD::*hrpL* exhibited low expression in the absence of arabinose and high expression in the presence of arabinose in minimal medium developed for repression of the native *hrpL* promoter (data not shown). Addition of 200 mM of arabinose to the medium was optimal for *hrpL* induction. Differential Fluorescence Induction (DFI) Vectors: Construction of DFI vectors was described in Chang et al. [26]. Briefly, pBBR1-MCS2 was modified to carry RNA terminators, and mutant GFP3, which has an excitation peak corresponding to the argon laser (488 nM) of the FACS. Three stop codons in each frame were annealed and cloned upstream from the Shine-Delgarno sequence as an EcoRI-XbaI fragment, yielding vector 125.1. PCR products were directly cloned into EcoRI-BamHI digested 125.1 vector following a sticky-end PCR protocol, resulting in DFI vectors.

### Library Construction for the Promoter-trap Screen

DNA was extracted from *P. carotovorum* WPP14, purified by using Epicentre MasterPure DNA extraction kit, and partially digested with either Tsp509I, or *Alu*I, *Bst*UI, *Hae*III, and *Rsa*I. Fragments from 1–1.6 kb, 1.6–3 kb, and 3–4 kb were extracted and cloned into either EcoRI- or *Sma*I-digested and shrimp alkaline phosphatase (SAP)-treated DFI vectors. *E. coli* colonies carrying approximately 66,000 library fragments were pooled and mated en masse by modified tri-parental mating with WPP14+ pBAD::*hrpL* and pRK2013.

### Promoter-trap Screen

The screen was done as described in Chang et al. [26]. Briefly, we analyzed the DFI library under the inducing conditions of 200 mM arabinose for 22 h before FACS screening. FACS was performed on a MoFlo (Cytomation) and analysis was performed on a FACscan from Becton Dickinson. One ml of culture grown over night in LB was pelleted and then diluted into 400µl of 1× PBS. HrpL-inducible gene fragments were identified with 4 subsequent sorts. The first, in the absence of induction, the least fluorescent cells (30%) were collected. These cells were grown in inducing conditions and a small population (less than 2% of total population) of highly-fluorescent cells was collected; these two sorts were repeated, except final fluorescent cells were collected in individual wells of a 364 well plate. Individual library clones were grown in these plates, their library fragments were amplified from the DFI vector, and amplicons were sequenced.

### Sequencing Library Clones

Approximately 2600 clones were sequenced. Candidate HrpL-induced gene fragments were amplified from cells harboring the DFI plasmids using *Taq* polymerase and primers, HYZ163 and HYZ166 [26]. PCR products were treated with 5 units of exonuclease I and 0.5 unit of SAP at 37°C for 40 min and heat terminated at 80°C for 30 min. Sequences were aligned to the WPP14 genome sequence.

### DspE Constructs

The GENEART® Site-Directed Mutagenesis system (Invitrogen) was used to create W464A, W514A and W660A mutations in *dspE* using pCH0001 for the template plasmid and sequence-specific primers (Tables S1 and S3) for each mutation. Double mutants W464A/W514A and W464A/W660A were created using pCH0007 for the template plasmid. The W514A/W660A double mutant was created using pCH0008 for the template plasmid and the triple mutant was created using pCH0016 for the template plasmid. All mutations were confirmed by DNA sequencing.

### Plant Growth Conditions

*A. thaliana* Col-0 were grown in 9 h day-length lighting conditions in a Conviron growth chamber at 23°C. *Nicotiana benthamiana* was grown in growth chambers at 26°C with a 12 h photoperiod under 40-W cool white fluorescent bulbs with photons at 240µmol/m^2^/s. *S. tuberosum* US-W4 and cv. “Dark Red Norland” plants used for agroinfiltration were grown from certified seed tubers in a greenhouse.

### Agroinfiltration Assays

*Agrobacterium* strains were grown for 48 h in a shaking incubator (200 rpm) at 28°C in 5 ml of LB medium supplemented with appropriate antibiotics. One ml of this culture was then added to 50 ml of fresh LB with appropriate antibiotics and then grown overnight in a shaking incubator (200 rpm) at 28°C. The culture was then centrifuged (10 min, 7200 ***g***) and resuspended in 5 ml of infiltration buffer (pH 5.7) (10 mM MgCl_2_, 10 mM MES pH5.6, 150µM acetosyringone). The bacterial suspension was diluted with the same buffer to adjust the inoculum concentration to an OD_600_ = 0.7. The *A. tumefaciens* inoculum was delivered to *N. benthamiana* and *S. tuberosum* leaves in the same manner described for *P. carotovorum* inoculum. The infected area of the leaf was delimited and labeled with an indelible pen. The treated plants were placed in a growth chamber under ambient humidity and reactions were recorded at 24 and 48 hpi. Each assay was performed in triplicate and all plant assays were repeated at least three times.

### Bacterial Sample Collection from Plants and RNA Isolation

Leaves of *N. benthamiana* were infiltrated with 1 ml of a cell suspension of *P. carotovorum* at OD_600_ = 1.0 made from an overnight culture grown on LBA and suspended in sterile water. For each strain three leaves were infiltrated, and RNA was isolated from each individually. Whole leaves were placed in cooled sterile RNase-free mortars pre-filled with 17.5 ml of DEPC water and 1.25 ml of ice cold EtOH/Phenol stop solution (5% water-saturated phenol, 95% EtOHl pH<7.0) to stabilize cellular RNA and stop degradation. Plant samples were gently ground with a sterile pestle. Supernatants were collected by pouring ground samples through a tea strainer and transferring the supernatant to two sterile 15 ml falcon tubes. Bacterial RNA was collected from supernatants according to the method described in Jahn et al. 2008 [27].

### Real-time RT-qPCR

Primers were designed based on the draft genome sequence of WPP14 using the Beacon Designer software (Premier Biosoft International, Palo Alto, CA, U.S.A). Primers were designed in regions of little secondary template structure. Sequence primers are shown in Table S2. Primer efficiency was determined using dilution series of target DNA [28]. RNA samples were tested for residual DNA contamination using qPCR and primers targeting the *proC* gene in WPP14. RNA samples that showed quantification cycle (Cq) values >30 cycles were considered to be sufficiently free of chromosomal DNA contamination.

cDNA synthesis was performed using iScript cDNA synthesis kit according to the manufacturer’s instructions (Bio-Rad Laboratories, Hercules, CA, U.S.A.). Briefly, cDNA synthesis was performed with 1 µg of total RNA in 16 µl of DEPC-water and 4 µl of 5X iScript reaction mix containing a blend of oligo(dT), random hexamer primers and reverse transcriptase. The reaction conditions were performed at 25°C for 5 m, 42°C for 30 m, and 85°C for 5 m. Three independent biological replicates samples per experimental condition were used for the cDNA synthesis. The cDNA reactions were diluted 10-fold and 24 µl was used for each reaction that was divided into three wells at 20 µl per well (technical replicates). The cDNA was quantified in the CFX-96 detection system (Bio-Rad Laboratories) using SsoFast EvaGreen Supermix (Bio-Rad Laboratories). PCR conditions were 98°C for 2 m; 40 cycles of 98°C for 2 s and 55°C for 5 s; followed by a dissociation curve with 80 cycles starting at 70°C for 10 s with a 0.2°C increase per cycle. Primer-dimers and presence of a single product per reaction were evaluated using the dissociation (Melt) curve analysis within the CFX detection system software (Bio-Rad Laboratories).

For relative expression analysis, target gene abundance was internally normalized to two reference gene transcripts (*recA* and *proC*) using the formula: 2^Δ*C*q(target-reference)^. The reference transcripts were found to be stable under all experimental conditions and were validated using the *BestKeeper* program [28]. The transcripts of five genes (*ffh*, *recA*, *proC*, *fliC*, and *gyrA*) were evaluated using the *BestKeeper* program. Of these, three transcripts (*recA*, *proC*, *and fliC*) were validated as stable reference transcripts under our experimental conditions. Target transcript amounts were reported as relative expression ratio (RER) of target transcript in the Δ*hrpL* strain relative to wild type. To determine the RER of target transcripts, the normalized target RNA value was divided by the average of the normalized values of wild-type samples. This average was designated as the “calibrator” since the variation of all samples, including the individual wild-type samples, was determined relative to this value. This method of calculating the RER was derived from the previously published 2^−ΔΔCq^ formula [28], [29]. Statistical analysis of RER values was conducted by the unpaired two-tailed t-test with 95% confidence interval using Prism 5.0a software (GraphPad Software, Inc.).

## Results

### Confocal Microscopy Confirms HrpL Activity in vivo

**Figure 1:**
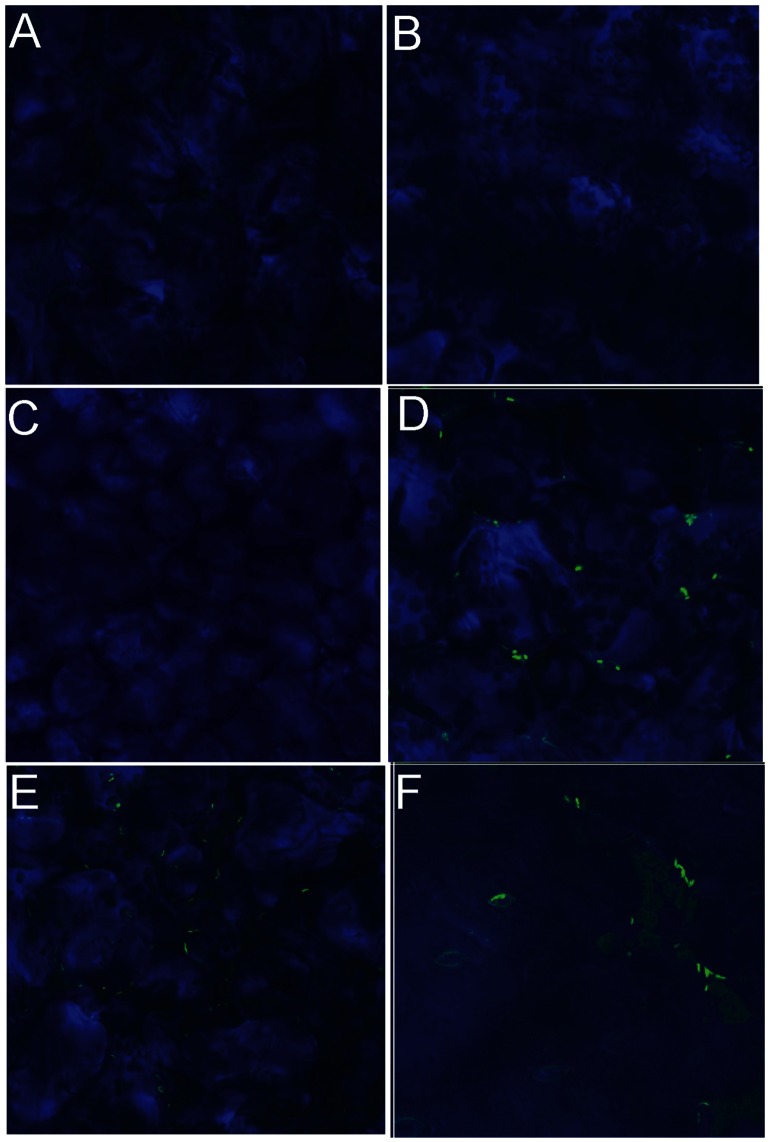
Confocal microscopy images at a magnification of 650X of *A. thaliana* Col-0 mesophyll cells 7 hours after infection with *P. carotovorum.* **A.** Mesophyll cells from an uninoculated leaf. **B.** Mesophyll cells from a leaf inoculated with WPP14. **C.** Mesophyll cells from a leaf inoculated with WPP14Δh*rpL*(pDFI::*dspE_pro_*GFP) as a negative control. **D.** Mesophyl cells from a leaf inoculated with 200 mM arabinose and WPP14(pDFI::*dspE_pro_*GFP) and pBAD::*hrpL* as a positive control. **E and F**. Mesophyl cells infected with WPP14 (pDFI::*dspE_pro_*GFP). Images are representative of three independent experiments in which at least one leaf was inoculated for each treatment.

We used confocal microscopy to visualize the activity of a HrpL-responsive reporter during leaf infection. Wild-type *P. carotovorum* WPP14 carrying a Differential Fluorescent Induction (DFI) vector [26] with a *dspE* promoter-*gfp* fusion construct was infiltrated into *Arabidopsis thaliana* Col-0. *A. thaliana* leaves were used due to their thinness relative to potato leaves. Leaves were visualized at 5 mins, 30 mins, 1 hour, 3 hours and 7 hours post infiltration, with peak fluorescence observed at 7 hours post infiltration. Later time-points were not examined with confocal microscopy due to leaf tissue maceration and for fear that the long half-life of GFP would skew interpretations of the timing of expression. Fluorescence, visualized 7 hours after infiltration, indicated native HrpL induction and activity, and T3SS expression *in vivo* (Fig. 1). When the *dspE_pro_*-*gfp* fusion was carried in a *hrpL* mutant, we did not see fluorescence at any of the sampled time points, indicating that the fluorescence was due to HrpL activity.

### Promoter-trap Screen Identifies HrpL-regulated Genes

We used a fluorescence activated cell sorter (FACS)-based promoter trap screen developed by Chang et al. [26] to identify T3SS effectors. Previous studies of the T3SS in *P. carotovorum* had identified the alternative sigma factor, HrpL, as the master regulator of the T3SS and the effector, DspE [14]. We exploited HrpL as a likely regulator of unidentified effectors and associated virulence factors and performed the FACS-based promoter trap screen as described by Chang et al. [26]. The *dspE* gene was cloned into the DFI vector [26] to serve as a positive control for induction, and peak fluorescence after *hrpL* induction. *dspE* and genome library fragments were inserted into the DFI vector and mobilized by tri-parental mating into *P. carotovorum* WPP14, which already carried *hrpL* on the arabinose-inducible pBAD plasmid. Cultures were sorted by FACS with 4 tandem sorts to enrich for *hrpL-*dependent fluorescing cells.

**Figure 2:**
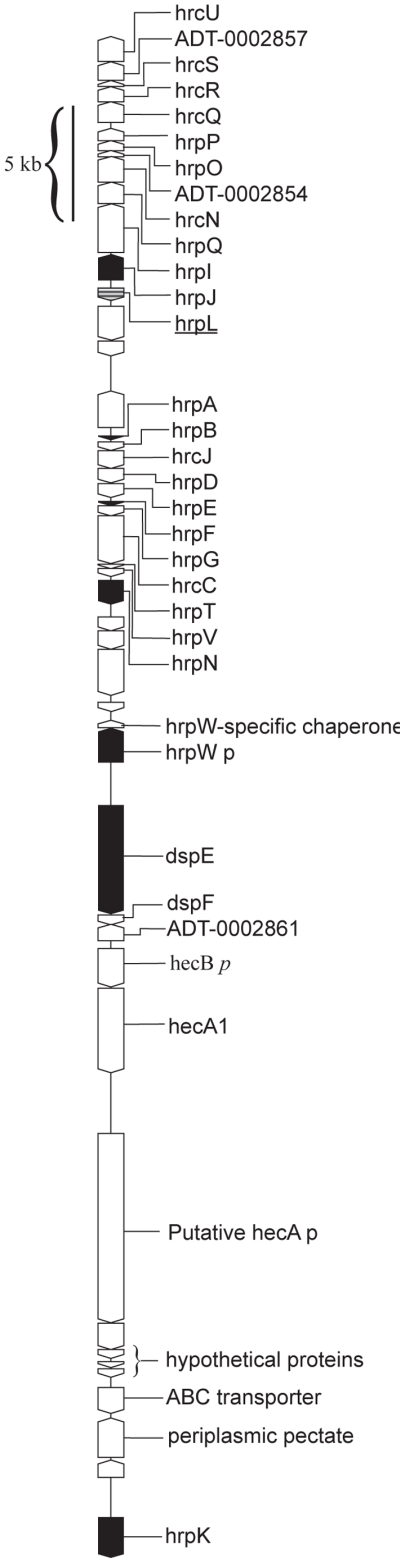
The Hrp pathogenicity island of *P. carotovorum* strain WPP14 (GenBank accession NZ_ABVT00000000). Black fill denotes detection in the fluorescence based promoter trap screen, and *p* indicates pseudogenes that have yet to be further examined. This figure was made from a draft genome sequence and it is likely that *hecA1* and putative *hecA* represent a single ORF.

Approximately 2600 clones were captured and their library fragments were sequenced and aligned to the genome. Sequences aligned to 37 distinct regions (<4 kb in length) of the genome. 17% of the sequences aligned to an arabinose operon responsive to the induction conditions and were therefore disregarded. Randomly selected clones representing each region were independently grown and re-screened by FACS to verify HrpL-dependent fluorescence. The verified clones represented 17 HrpL-inducible regions and a full length ORF was identified in each region. To confirm that these regions were regulated by HrpL, we used qRT-PCR to analyze expression of ORF’s contained in these 17 regions in a WPP14Δ*hrpL m*utant strain carrying *hrpL* fused to an arabinose inducible promoter under inducing conditions. Only 6 genome regions (representing 58% of the sequenced library clones) were confirmed with qRT-PCR, and these all aligned to known HrpL-dependent genes and operons within the T3SS gene cluster (Table 1, Fig. 2).

**Table 1:** HrpL-regulated genes found in the DFI screen.

Gene	ASAP ID#	Function	Number of Clones	Minimum number of independent inserts
*hrpQ/J*	ADT-0002852	Type III secretion system component	784	74
*hrpA*	ADT-0001735	Type III secretion system component	5	2
*hrpF*	ADT-0001736	Type III secreted protein	6	2
*hrpW*	ADT-0002859	Type III secreted protein	149	16
*hrpK*	ADT-0002735	Type III secretion system component	196	17
*dspE*	ADT-0003863	Type III secreted effector	329	19

T3SS accessory genes are involved in apparatus assembly. When a *hrp* cluster operon was identified, the first gene of the operon is listed.

The region that was most frequently identified, represented by 784 clones, was a 12 member operon within the T3SS cluster. Known *hrp* cluster members and predicted HrpL binding sites were based on a published description of the T3SS cluster [13], [16], [30]. *dspE*, the only previously identified effector protein, was represented in the screen 329 times. *hrpA*, encoding a T3SS-associated pilin, and *hrpF*, a putative T3SS translocon, were represented the least, with only 6 and 4 representative clones, respectively. Only one known HrpL-regulated gene was not identified in the screen, *hrpN*. We cloned *hrpN* into the DFI vector to assess its fluorescent profile. We found that *hrpN* expression was inducible via HrpL; however its basal expression level was higher than 30% of the fluorescence of the library, suggesting the reason it was not captured during the screen (data not shown).

### Expression of *dspE* and *hrpN* Requires *hrpL in Planta*

**Figure 3:**
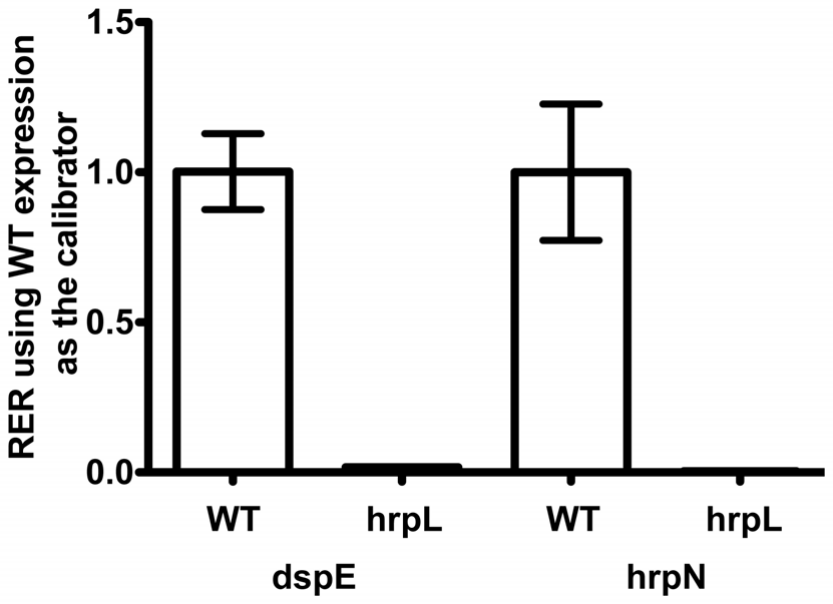
*In planta* expression of select *P. carotovorum* genes *dspE* and *hrpL* at 5 hpi. Relative expression of the *dspE* and *hrpL* transcripts in *A. thaliana* Col-0 leaves was determined with two validated reference mRNA (*recA* and *proC* - Table S2). The mean relative expression ratio (RER) of the *dspE* and *hrpN* transcripts in the *hrpL* mutant was 0.017 and 0.003, respectively, compared to expression in the wild type strain. The mean normalized transcript abundance relative to reference mRNA was used as the calibrator. The difference in the RER means in the *hrpL* mutant compared to the wild type for both target transcripts was found to be significant at a 95% confidence interval by Anova with a Tukey Post Test using Prism software (Mac version 5.0c, GraphPad Software, Inc.). Error bars represent standard error of the mean.

Previously, we found that *hrpL* and *dspE*, but not *hrpN* or *hrpW*, are required for disease and necrosis on plant leaves [11]. To verify the *in planta* dependence of T3SS gene expression on HrpL shown by our *dspE_pro_*-*gfp* fusion analysis, we used real-time reverse transcriptase quantitative PCR (real-time RT-qPCR) to compare *dspE* and *hrpN* expression in wild type *P. carotovorum* WPP14 versus a Δ*hrpL* strain. Leaves of *N. benthamiana* were inoculated with either the wild type or mutant strain with a needleless syringe. Total RNA was extracted from infected leaves five hours post-inoculation, which is the time when leaves infiltrated with the wild-type strain consistently began to wilt at this time point, indicating activation of pathogenesis. Expression of *hrpN* and *dspE* relative to the validated reference genes *recA* and *pro*C were quantified for each strain. Both genes were expressed in leaves and expression of both *dspE* and *hrpN* was reduced more than 59-fold in the Δ*hrpL* mutant at 5 hours post inoculation (Fig. 3). In both cases, the target transcripts were produced in the *hrpL* mutant near the limit of detection for real-time RT-qPCR.

### DspE alone is able to Elicit Cell Death in Leaf Tissue

**Figure 4:**
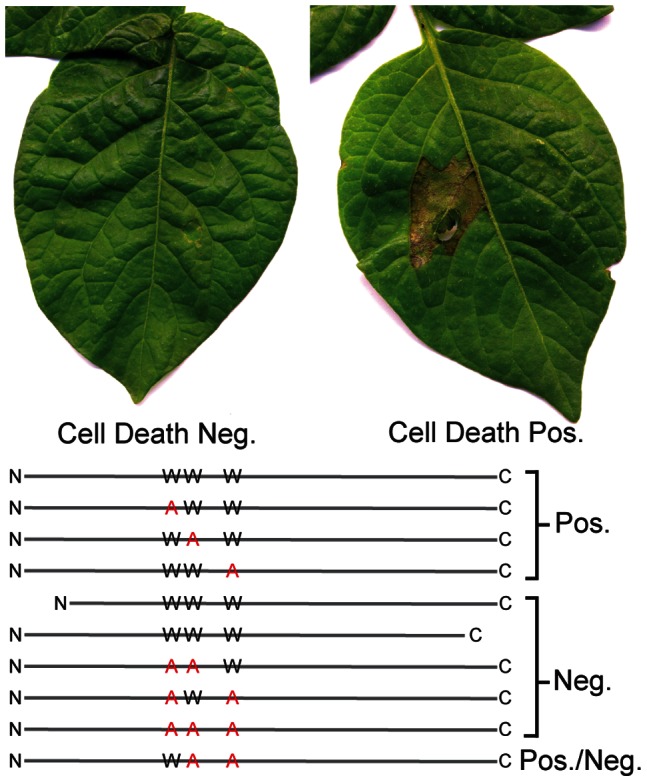
Heterologously expressed DspE causes cell death. Deletion of either the N- or C- terminus as well as certain tryptophan-to-alanine substitutions inhibit function. *Agrobacterium* mediated transformation of *S. tuberosum* leaves for expression of DspE and a series of mutant constructs. These leaf images are typical of a positive or negative cell death response. Combination of any two of the W to A substitutions reduced or eliminated function. The derivative containing only the two most downstream substitutions caused an inconsistent cell death response.

To study the activity of DspE in the absence of other effector proteins produced by *P. carotovorum* WPP14, we transiently expressed *dspE* in *N. benthamiana* leaves by agroinfiltration [31] and verified these results in *Solanum tuberosum* leaves [32]. Infiltration of *Agrobacterium tumefaciens* carrying the wild-type *dspE* clone (pCH0002) resulted in necrotic lesions that resembled the hypersensitive response (HR) in both *N. benthamiana* and *S. tuberosum* 48 hours post inoculation (Fig. 4). These data establish that DspE is sufficient for HR elicitation and complement our previous work where deletion of *dspE* eliminates the ability of *P. carotovorum* WPP14 to kill leaf tissue [11].

### Deletion Analysis and Site-directed Mutagenesis Revealed that the Entire Length of DspE, and Wx_(3–6)_D/E Motifs are Required for Cell Death

We precisely deleted the N-terminal 330 amino acids and the C-terminal 98 amino acids from DspE. When expressed in *N. benthamiana* leaves, these mutant proteins did not cause necrosis (Fig. 4), suggesting that these deletions destabilize the protein or contain motifs important for function.

**Figure 5:**
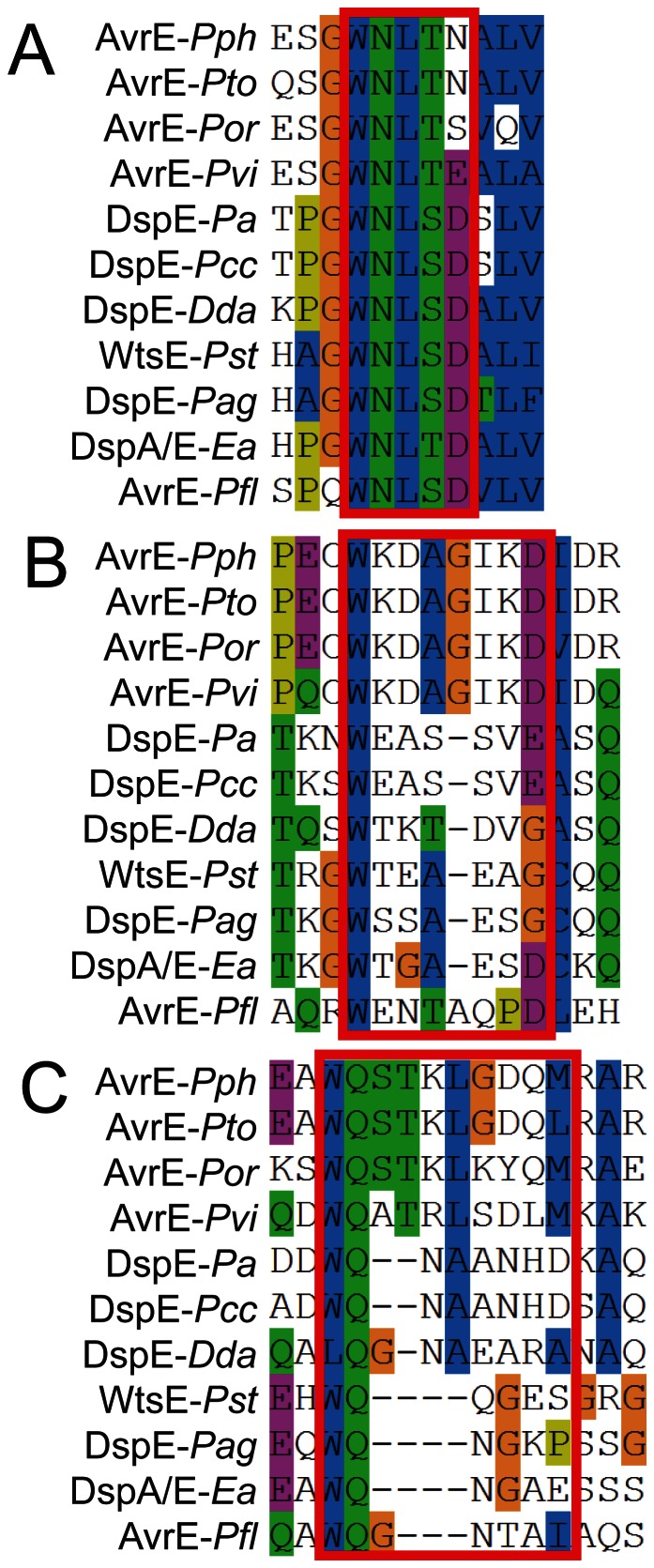
Multiple sequence alignment with hierarchical clustering of DspE homologs in plant pathogenic bacteria. **A.** W464, **B.** W514, **C.** W660 in *P. carotovorum* WPP14 protein sequence. Dashes indicate gaps. Genus and species abbreviations and protein names are as follows *Pph* (*Pseudomonas syringae* pv. *phaseolicola* 1448A, NCBI Ref. Seq. YP_273527.1), *Pto* (*Pseudomonas syringae* pv. *tomato* DC3000, NCBI Ref. Seq. NP_791204.1), *Por* (Pseudomonas syringae pv. oryzae, NCBI Ref. Seq. ZP_04588443.2), *Pvi* (*Pseudomonas viridiflava*, NCBI Ref. Seq. GenBank accession AAT96164.1), *Pa* (*Pectobacterium atrosepticum* SCRI1043, GenBank accessionYP_050208.1), *Pcc* (*Pectobacterium carotovorum* subsp. *carotovorum* WPP14, NCBI Ref. Seq. ZP_03833468.1), *Dda* (*Dickeya dadantii* 3937, NCBI Ref. Seq. YP_003883118.1), *Pst* (*Pantoea stewartii* subsp. *stewartii*, GenBank accession AAG01467.2), *Pag* (*Pantoea agglomerans* pv. *gypsophilae,* GenBank accession AAF76343.1), *Ea* (*Erwinia amylovora*, GenBank accession AAC04850.1), *Pfl* (*Pseudomonas fluorescens*, GenBank accession AAK74145.1).

Our searches of the predicted *P. carotovorum* WPP14 DspE sequence revealed only one WxxxD motif at position 464. However, there are highly conserved tryptophans within 6 residues of an aspartic acid (D) or glutamic acid (E) that aligned to WxxxE motifs found in WtsE at DspE positions W514 and W660 (Fig. 5). Changing either the W or E residue to A in both motifs in the DspE homologs of *Pseudomonas syringae* (AvrE) and *Pantoea stewartii* (WtsE) resulted in loss of cell killing activity, as long as both WxxxE motifs contained at least one A substitution [33]. Therefore, we created a series of DspE mutants with various combinations of W to A substitutions at the sites mentioned above. These mutations were constructed by site-directed mutagenesis in plasmid pCH0001. The single mutants were designated DspE-w1 (W464A), DspE-w2 (W514A) and DspE-w3 (W660A). The double mutants were designated DspE-w12, DspEw13 and DspE-w23, and the triple mutant was designated DspE-w123. We found that, like AvrE and WtsE, all of the DspE mutants containing a single substitution retained cell-killing activity (Fig. 4). However, the w12, w13, w23 and w123 mutants failed to induce necrosis (Fig. 4), though in one of five assays the w23 mutants would show attenuated leaf cell death (data not shown). We increased the expression level of these mutants by co-expressing the *Tobacco etch virus* silencing suppressor P1/HC-Pro [34]. Co-expression of wild type DspE with HC-Pro caused necrosis one-day post inoculation, compared to 2 days without HC-pro, suggesting that an increase in the expression level of the protein resulted in a swifter reaction. It is also possible that the presence of the silencing suppressor affected the leaf sensitivity to DspE and derivatives. Co-expression of w23 with HC-Pro caused necrosis to consistently appear two days post-inoculation, indicating that the combination of w2 and w3 mutations partially attenuated cell-killing function of the protein, rather than completely eliminating this function.

As with a previous report on WtsE [33], attempts to monitor *in planta* production and stability of DspE following *Agrobacterium*-mediated transient gene expression failed to detect DspE or its derivatives (data not shown). However, mutations of two or three amino acids in the w12, w13, w23 and w123 mutant alleles are unlikely to affect expression or stability of DspE, since each mutation alone had no effect on elicitation of plant necrosis. Additionally, the ability of the w23 DspE mutant allele to cause cell death when expression is increased by the presence of HC-Pro indicates that the mutant protein accumulates and retains attenuated function in leaf cells. Furthermore, similar mutations in DspE homologs did not affect production or secretion [33]. Together, these data indicate that Wx_(3–6)_D/E motifs are functionally redundant, but required for DspE mediated host cell death.

### HrpL is Required for the Expression of Genes Outside of T3SS Cluster *in Planta*

**Figure 6:**
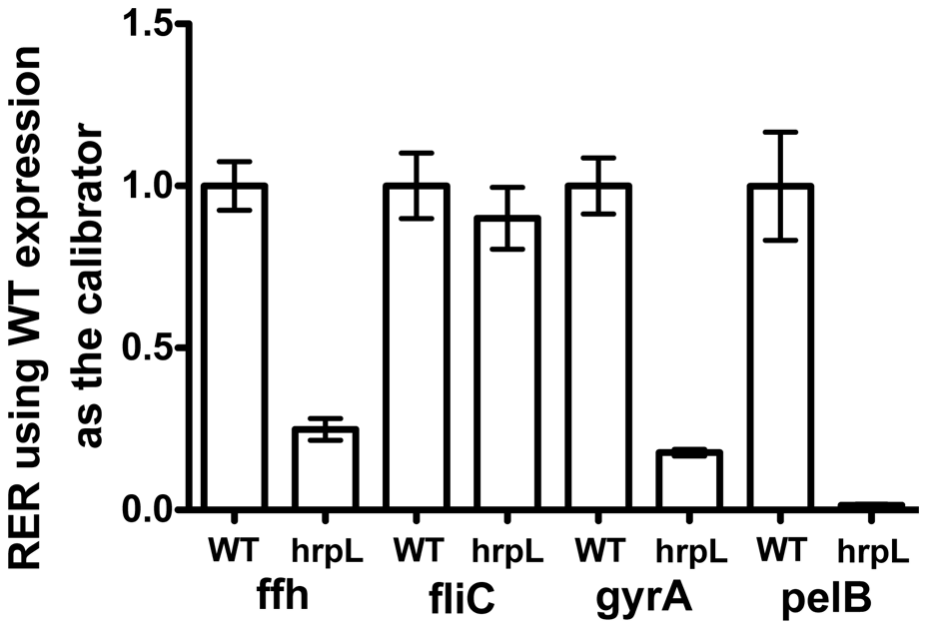
*In planta* expression of select *P. carotovorum* genes *dspE* and *hrpL* at 5 hpi. Relative expression of the *ffh*, *fliC*, *gyrA* and *pelB* transcripts in *A. thaliana* Col-0 leaves was determined with two validated reference mRNA (*recA* and *proC* - Table S2). The mean relative expression ratio (RER) of the *ffh*, *fliC*, *gyrA* and *pelB* transcripts in the *hrpL* mutant was 0.25, 0.90, 0.18 and 0.015, respectively, compared to expression in the wild type strain. The mean normalized transcript abundance relative to reference mRNA was used as the calibrator. The difference in the RER means in the *hrpL* mutant compared to wild-type for all target transcripts except *fliC* was found to be significant at a 95% confidence interval by Anova with a Tukey Post Test using Prism software (Mac version 5.0c, GraphPad Software, Inc.). Error bars represent standard error of the mean.

In addition to the T3SS genes, we examined the effect of the Δ*hrpL* mutation on the expression of several other *P. carotovorum* genes *in planta*. Surprisingly, several of these genes were also attenuated in the mutant. The *ffh* and *gyrA* genes are conserved among Gram-negative bacterial species and are often used as reference transcripts for real-time RT-qPCR in *Pectobacterium* species [35]. In the course of validating reference transcripts for bacteria infiltrated into leaves, we found that expression of *ffh* was reduced 4-fold and *gyrA* was reduced 5.5-fold in the Δ*hrpL* mutant (Fig. 6). Since *P. carotovorum* T3SS mutants do not macerate leaves, we also examined the expression of *pelB,* which encodes pectate lyase, and found it to be reduced 67-fold in the Δ*hrpL* mutant. Expression of a flagellin gene, *fliC,* was not affected (Fig. 6), which was unexpected based on previously reported results in *Erwinia amylovora* [36].

## Discussion

We found that *P. carotovorum* subsp. *carotovorum* strain WPP14 (*P. carotovorum* WPP14) activates the T3SS during the early stages of infection of leaf tissue and appears to translocate a single effector, DspE. Nearly all of the genes regulated by HrpL are T3SS genes or homologous to known secreted proteins, including *hrpN*, *hrpK*, *hrpW*, and *dspE*. The *hrpK* gene is separated from the rest of the T3SS gene cluster by insertion of an operon homologous to *hecAB* (Fig. 2).

We also found that *P. carotovorum* DspE is sufficient for killing both tobacco and potato leaf cells when expressed transiently in plant cells. Multiple WxxxD/E motifs are required by *P. carotovorum* DspE for cell-killing activity (Fig. 4), as has been found with homologs from other bacterial species [33]. These data support findings from previous studies in which dspE was the only effector homolog identified by sequence analysis and deletion of either *dspE* or *hrpL* eliminated the ability of *P. carotovorum* to cause leaf cell death [11], [13].

We confirmed that HrpL, an alternative sigma factor required for expression of the T3SS and other pathogenesis associated genes [11], [37], is required for expression of the T3SS genes *dspE* and *hrpN* in plant leaves. Interestingly, the expression of several genes outside of the T3SS cluster is reduced in leaves in our *hrpL* mutant, including *pelB* and the central metabolism genes *ffh* and *gyrA* (Fig. 6). Since no HrpL binding site is evident upstream of these genes, this suggests that plant responses to a functional T3SS affect expression of a wide range of genes not directly regulated by HrpL. Down-regulation of *pelB* is consistent with the no-maceration phenotype we see with T3SS mutants or strains naturally lacking a T3SS when they are infiltrated into plant leaves [11], [38]. The down-regulation of central metabolism genes *ffh* and *gyrA* highlights the importance of testing reference genes under the conditions being studied, rather than relying on previously reported reference genes in new experimental conditions.

Cell death is required for *P. carotovorum* WPP14 pathogenesis in leaves and depends upon the HrpL-regulated T3SS and *dspE/F* [11], [38]. We showed, through heterologous gene expression, that DspE alone is able to cause plant cell death (Fig. 4). Whether cell death is due to direct toxicity of the DspE protein, and/or because DspE function triggers plant programmed cell death, remains unknown. The hypothesis that DspE is recognized by a host R protein that subsequently activates the hypersensitive response (HR) is supported by the requirement for SGT1 for *P. carotovorum*-mediated leaf cell death [39]. Further supporting this hypothesis is the observation that DspA/E from *Erwinia amylovora* interacts with several leucine-rich repeat (LRR) receptor-like serine threonine kinases (RLK) [40]. Additionally, *P. carotovorum* is unable to infect *Arabidopsis* “defense, no death” mutants [41], suggesting that elicitation of leaf cell death is required for *P. carotovorum* pathogenesis. Taken together, this evidence supports a model in which DspE aids the necrotrophic *P. carotovorum* in attacking leaf tissue by eliciting R-protein mediated cell death.

Alternatively, DspE may activate programmed cell death by using WxxxE/D motifs to mimic a plant guanine nucleotide exchange factor (GEF), a class of proteins that regulate many processes in the cell including the HR [42]. Other AvrE-family proteins from hemibiotrophic pathogens possess one or two W/YxxxE/D motifs [33] and deletion of these motifs eliminates the plant cell death activity of AvrE from *Pseudomonas syringae* and WtsE from *P. stewartii*. This motif is also commonly found in animal pathogen T3 effectors, where these WxxxE-containing effectors are thought to act as regulators of the Rho family of GTPases by functioning as GEFs [43]. Rho GTPases are important regulators of many cellular processes including vesicle trafficking and apoptosis; processes that are important for pathogen resistance [44]. Thus, disruption of Rho GTPase signaling using type III effectors is an effective strategy for pathogens to modulate the host response. These two hypotheses, activation of R-protein mediated plant cell death and mimicking a GEF protein, are not mutually exclusive. The relative importance of each pathway to virulence may depend upon the physiological state of the host or the plant species infected by this broad host range pathogen.

We found that DspE relies upon three functionally redundant motifs consisting of a tryptophan (W), and either an aspartic (D) or glutamic acid (E) separated by a spacer region of three to six amino acids (Fig. 5). When the W from at least two of these motifs is substituted with an alanine residue, the cell killing ability of DspE is abolished, though single amino acid substitutions have no effect. The double substitutions are unlikely to affect stability of DspE since each mutation alone had little or no effect on cell killing activity. Furthermore, similar mutations in DspE homologs did not affect production or secretion [23]. These motifs closely resemble WxxxE motifs important for function of DspE homologs AvrE from *Pseudomonas syringae* DC3000 and WtsE from *Pantoea stewartii* (Fig. 5), which, like DspE, elicit plant cell death [33]. Further supporting this hypothesis is the observation that AvrE and WtsE suppress callose deposition and have an endoplasmic reticulum membrane retention signal (ERMRS) [33]. These features indicate that AvrE and WtsE interfere with vesicle trafficking, a process known to be regulated by GEFs.

Notably, unlike AvrE and WtsE, *P. carotovorum* DspE lacks both callose deposition suppressing activity and an ERMRS [11], indicating that it may lack GEF activity. It is possible that the cell death activity shared between AvrE, WtsE and DspE is caused by a mechanism independent from callose deposition suppressing activity and does not require an ERMRS. Crystal structure analyses of the animal pathogen WxxxE effectors suggest a structural, instead of a catalytic, role for the WxxxE motif [43], [45]. Thus, while mutation of the WxxxE motif may result in a loss of GEF activity due to destabilization of a catalytic site, the loss of cell death activity may be attributed to a conformational change that disrupts recognition of the effector or its activities by an R protein. Further studies are needed to determine whether DspE possesses GEF activity, whether AvrE or DspE affects vesicular trafficking, and which host proteins interact with DspE.

It appears that acquisition of a T3SS and an AvrE-family effector allowed *P. carotovorum* to expand the range of tissues that it can infect to include plant leaves. The biochemical function of DspE remains unknown, but the lack of an ERMRS and callose suppressing activity suggest that *P. carotovorum* requires only the ability to induce host cell death in order to infect leaf tissue and that this is achieved by maintaining a single effector protein that is active in a wide range of plant species. In the case of *P. carotovorum* WPP14, this effector is DspE, although it is possible that other strains use other effectors. Sequence analysis, promoter trap analysis, and mutational analysis all support the hypothesis that WPP14 encodes only a single effector, DspE, and genome sequence analysis of other *Pectobacterium* strains has yet to identify additional putative T3 effectors [13], [46]. Additional biochemical characterization of DspE and analysis of *P. carotovorum* ecology is needed to further support this model.

## Supporting Information

Table S1 **Plasmids and bacterial strains used in this study.**
(DOCX)

Table S2 **Real-time RT-qPCR primer sequences and efficiency.**
(DOCX)

Table S3 **Primers for cloning DspE and derivatives.**
(DOCX)
