# Supplementary material for: The Type III Secreted Effector DspE Is Required Early in Solanum tuberosum Leaf Infection by Pectobacterium carotovorum to Cause Cell Death, and Requires Wx(3–6)D/E Motifs
Source: PLoS One. 2013 Jun 3;8(6):e65534. doi: 10.1371/journal.pone.0065534 (PMC3670860; doi:10.1371/journal.pone.0065534)
Supplement: Table S1 — Plasmids and bacterial strains used in this study. (DOCX) [file pone.0065534.s001.docx]

**Table S1.** Plasmids and bacterial strains used in this study

| **Plasmids and Strains** | **Description^a^** | **Source or reference** |
| --- | --- | --- |
| *Agrobacterium tumefaciens* |  |  |
| GV3101 | Vir-, Gm^r^, Rif^r^ | Van Larebeke et al. 1974 |
| C58C1­^D^ | Vir-, Gm^r^, Rif^r^ | Johansen and Carrington, 2001 |
| *Escherichia coli* |  |  |
| DH5α-E™ | F- φ80*lac*Z∆M15 ∆(*lac*ZYA-*arg*F) U169 *rec*A1 *end*A1 *hsd*R17 (rk-, mk+) *gal*- *pho*A *sup*E44 λ- *thi*-1 *gyr*A96 *rel*A1 | Invitrogen Corp |
| DH5α™-T1^R^ | F- φ80*lac*ZΔM15 Δ(*lac*ZYA-*arg*F)U169 *rec*A1 *end*A1 *hsd*R17(rk-, mk+) *pho*A *sup*E44 *thi*-1 *gyr*A96 *rel*A1 *ton*A (confers resistance to phage T1) | Invitrogen Corp |
| *Pectobacterium carotovorum* subsp. *carotovorum* |  |  |
| WPP14 | Wild-type strain isolated from an infected potato stem from a Wisconsin field in 2001 | Ma et al. 2007; Yap et al. 2004 |
| WPP477 | Sp^r^, Δ*dspE/F*::Sp derivative of WPP14 | Kim et al. 2011 |
| WPP500 | Cm^r^, Δ*hrpL*::Cm derivative of WPP14 | This work |
| **PLASMIDS** |  |  |
| pCFS40 | Tet^r^ , contains MCS downstream of arabinose inducible promoter | Chang et al. 2005 |
| pBAD::*hrpL*WPP14 | Tet^r^, contains *hrpL* under control of an arabinose inducible promoter | This work |
| pBBR1-MCS2 | Km^r^, broad host range cloning vector | Kovach et al. 1995 |
| pRK2013 | Km^r^, broad host range triparental mating helper plasmid | Figurski and Helinski, 1979 |
| 125.1 | Km^r^, DFI vector, derivative of pBBR1-MCS2 | This work |
| pCR^®^2.1-TOPO^®^ | Amp^r^, Km^r^, subcloning vector | Invitrogen Crop |
| pCR2.1SOE*hrpL* | Amp^r^, Km^r^, contains 1kb regions flanking WPP14 *hrpL* | This work |
| pCR2.1∆hrpL | Amp^r^, Cm^r^, Km^r^, contains Cm^r^ cassette inserted between WPP14*hrpL* flanking regions | This work |
| pDONR221 | Km^r^ A donor vector for the Gateway^®^ cloning system | Invitrogen Corp |
| pGWB2 | Km^r^, Hy^r^, Binary vector for agroinfiltration and  *in planta* expression under control of the *Cauliflower mosaic virus* 35s promoter | Nakagawa et al., 2007 |
| pCH0001 | Km^r^ pDONR221::*dspE* from *P. carotovorum* subsp. *carotovorum* strain WPP14 | This work |
| pCH0002 | Km^r^, Hy^r^, pGWB2::*dspE* from *P. carotovorum* subsp. *carotovorum* strain WPP14 | This work |
| pCH0003 | Km^r^ pDONR221::*dspE-*Δaa1-330 | This work |
| pCH0004 | Km^r^ pDONR221::*dspE-*Δaa1529-162 | This work |
| pCH0005 | Km^r^, Hy^r^, pGWB2::*dspE-*Δaa1-330 | This work |
| pCH0006 | Km_­_^r^, Hy^r^, pGWB2::*dspE-*Δaa1529-162 | This work |
| pCH0007 | Km^r^, Derivative of pCH0001 with DspE-W464A | This work |
| pCH0008 | Km^r^, Derivative of pCH0001 with DspE-W514A | This work |
| pCH0009 | Km^r^, Derivative of pCH0001 with DspE-W660A | This work |
| pCH0010 | Km^r^, Derivative of pCH0001 with DspE-W464A/W514A | This work |
| pCH0011 | Km^r^, Derivative of pCH0001 with DspE-W464A/W660A | This work |
| pCH0012 | Km^r^, Derivative of pCH0001 with DspE-W464A/W514A/W660A | This work |
| pCH0013 | Km^r^, Hy^r^, pGWB2::*dspE*-W464A | This work |
| pCH0014 | Km^r^, Hy^r^, pGWB2::*dspE* -W514A | This work |
| pCH0015 | Km^r^, Hy^r^, pGWB2::*dspE* -W660A | This work |
| pCH0016 | Km^r^, Hy^r^, pGWB2::*dspE* -W464A/W514A | This work |
| pCH0017 | Km^r^, Hy^r^, pGWB2::*dspE* -W464A/W660A | This work |
| pCH0018 | Km^r^, Hy^r^, pGWB2::*dspE* -W464A/W660A | This work |
| pCH0019 | Km^r^, Hy^r^, pGWB2::*dspE* -W464A/W514A/W660A | This work |
| pSLJ755I5::HC-Pro_TEV_ | Km^r^ | Johansen and Carrington, 2001 |

^a^Amp^r^, Cm^r^, Gm^r^, Rif^r^,Sp^r^, Km^r^, Hy^r^ indicate resistant to ampicillin, chloramphenicol, gentamycin, rifampicin, spectinomycin, kanamycin and hygromycin, respectively. Appropriate antibiotics were added at the following concentrations: ampicillin (100 µg/ml), chloramphenicol (30 µg/ml) gentamycin (50µg/), hygromycin (50 µg/ml) kanamycin (50 µg/ml) rifampicin (15 µg/mL for *A. tumefaciens*, spectinomycin (50 µg/ml), and tetracycline (5 µg/ml).
