# Supplementary material for: The Type III Secreted Effector DspE Is Required Early in Solanum tuberosum Leaf Infection by Pectobacterium carotovorum to Cause Cell Death, and Requires Wx(3–6)D/E Motifs
Source: PLoS One. 2013 Jun 3;8(6):e65534. doi: 10.1371/journal.pone.0065534 (PMC3670860; doi:10.1371/journal.pone.0065534)
Supplement: Table S2 — Real-time RT-qPCR primer sequences and efficiency. (DOCX) [file pone.0065534.s002.docx]

**Table S2**. Real-time RT-qPCR primer sequences and efficiency

| **Gene Name** | **Primer Sequence** | **Efficiency (%)^A^** |
| --- | --- | --- |
| *ffh* | Forward: 5’-TGGAAACATTGGCAGAGC-3’  Reverse: 5’-GACTAACAAGACATCGTAGAAC-3’ | 105 |
| *gyrA* | Forward: 5’- CGTGAAACCATCATCGTG-3’  Reverse: 5’-TTAACCAGCTCGGCAATT-3’ | 95 |
| *proC* | Forward: 5’-GTGCGAATTATGCCAAAC-3’  Reverse: 5’-TTATCTGCCTGACTGACG-3’ | 100 |
| *recA* | Forward:5’-GTAACCCTGAAACGACTA-3’  Reverse: 5’CAGTACGACGAATATCCA-3’ | 92 |
| *pelB* | Forward: 5’-CTCCGTAACAACAACATT-3’  Reverse: 5’-GTACTCTTCCAGTCATCT-3’ | 102 |
| *fliC* | Forward: 5’-CGAATCTACCATTACTAACCT-3’  Reverse: 5’-AGTCAGCGTCTTCAATAC-3’ | 99 |
| *dspE* | Forward: 5’-GTCCTATACCAACCTCAG-3’  Reverse: 5’-GCAACGAAGAGAACAAAT-3’ | 99 |
| *hrpN* | Forward: 5’-CAGGAGTTGAACAACATTAG-3’  Reverse: 5’-CCATCTTACGGTCTTCTT-3’ | 89 |
| *hrpL* | Forward: 5’-AATGACCTATCTGGAAGT-3’  Reverse: 5’-TTGAAATAATTGCGAACC-3’ | 95 |

^A^Primer efficiencies were calculated from dilution curve using MyiQ Cycler software.
