# Supplementary material for: The Type III Secreted Effector DspE Is Required Early in Solanum tuberosum Leaf Infection by Pectobacterium carotovorum to Cause Cell Death, and Requires Wx(3–6)D/E Motifs
Source: PLoS One. 2013 Jun 3;8(6):e65534. doi: 10.1371/journal.pone.0065534 (PMC3670860; doi:10.1371/journal.pone.0065534)
Supplement: Table S3 — Primers for cloning DspE and derivatives. (DOCX) [file pone.0065534.s003.docx]

**Table S3. Primers for cloning DspE and derivatives**

| **Primer Name** | **Description** | **Sequence** |
| --- | --- | --- |
| DspE-1f | Sequencing forward primer starting at nt 1nt of *dspE* | ATGCAGAAAATCCAGCAT |
| DspE-985f | Sequencing forward primer starting at nt 991 of *dspE* | TTTTCGGCCAATCAGAAC |
| DspE-4884r-I | Sequencing reverse primer starting at nt 4890 of *dspE* | TTAACCGTTAAGCTGTAG |
| DspE-1f-I | First step forward adapter primer for cloning *dspE* with Gateway® attB1 site | GGCTTCGAAGGAGATAGAACCATGCAGAAAATCCAGCATGTT |
| DspE-985f-I | First step forward adapter primer for cloning *dspE*Δ1-330aa with Gateway® attB1 site | GGCTTCGAAGGAGATAGAACCATG |
| DspE F-II universal | Second step adapter primer for cloning *dspE* with Gateway® attB1 site | GGGGACAAGTTTGTACAAAAAAGCAGGCTTCGAAGGAGATAGA |
| DspE-4589r-I | First step reverse adapter primer for cloning *dspE*Δ1530-1628aa with Gateway® attB2 site | GTACAAGAAAGCTGGGTCCTATGCCGTGCGTCT |
| DspE-4589r-II | Second step reverse adapter primer for cloning dspEΔ1530-1628aa with Gateway® attB2 site | GGGGACCACTTTGTACAAGAAAGCTGGGTCCTATGC |
| DspE-4905r-I | First step reverse adapter primer for cloning *dspE* with Gateway® attB2 site | GTACAAGAAAGCTGGGTCCTAATCACCGCGTGTTAT |
| DspE-4905r-II | Second step reverse adapter primer for cloning *dspE* with Gateway® attB2 site | GGGGACCACTTTGTACAAGAAAGCTGGGTCCTAATC |
| DspE-W464A-f | Forward primer for tryptophan to alanine substitution at position 464 in DspE protein | TCGCCAACGCCGGGGGCAAACCTGAGCGACAGT |
| DspE-W464A-r | Reverse primer for tryptophan to alanine substitution at position 464 in DspE protein | ACTGTCGCTCAGGTTTGCCCCCGGCGTTGGCGA |
| DspE-W514A-f | Forward primer for tryptophan to alanine substitution at position 514 in DspE protein | AACGGCAATACCAAAAGCGCAGAGGCATCCAGCGTTGAA |
| DspE-W514A-r | Reverse primer for tryptophan to alanine substitution at position 514 in DspE protein | TTCAACGCTGGATGCCTCTGCGCTTTTGGTATTGCCTTG |
| DspE-W660A-f | Forward primer for tryptophan to alanine substitution at position 660 in DspE protein | CTGCCGAAAGCCGACGCACAAAATGCGGCGAAT |
| DspE-W660A-r | Reverse primer for tryptophan to alanine substitution at position 660 in DspE protein | ATTCGCCGCATTTTGTGCGTCGGCTTTCGGCAG |
